# Consumers in the Face of COVID-19-Related Advertising: Threat or Boost Effect?

**DOI:** 10.3389/fpsyg.2022.834426

**Published:** 2022-03-07

**Authors:** Michela Balconi, Martina Sansone, Laura Angioletti

**Affiliations:** ^1^International Research Center for Cognitive Applied Neuroscience (IrcCAN), Università Cattolica del Sacro Cuore, Milan, Italy; ^2^Research Unit in Affective and Social Neuroscience, Department of Psychology, Università Cattolica del Sacro Cuore, Milan, Italy

**Keywords:** emotional engagement, COVID-19, advertising, consumer neuroscience, neuromarketing, fNIRS

## Abstract

The COVID-19 pandemic has prompted the production of a vast amount of COVID-19-themed brand commercials, in an attempt to exploit the salience of the topic to reach more effectively the consumers. However, the literature has produced conflicting findings of the effectiveness of negative emotional contents in advertisings. The present study aims at exploring the effect of COVID-19-related contents on the hemodynamic brain correlates of the consumer approach or avoidance motivation. Twenty Italian participants were randomly assigned to two different groups that watched COVID-19-related or non-COVID-19-related commercials. The hemodynamic response [oxygenated (O_2_Hb) and deoxygenated hemoglobin modulations] within the left and right prefrontal cortices (PFC) was monitored with Functional Near-Infrared Spectroscopy (fNIRS) while brand commercials were presented, as the prefrontal lateralization was shown to be indicative of the attitude toward the brand and of the approach-avoidance motivation. First, the findings showed that the COVID-19-related contents were able to prompt emotional processing within the PFC to a higher extent compared to contents non-related to COVID-19. Moreover, the single-channel analysis revealed increased O_2_Hb activity of the left dorsolateral PFC compared to the left pars triangularis Broca’s area in the group of participants that watched the COVID-19-related commercials, suggesting that the commercials may have driven participants to dedicate more attention toward the processing of the emotional components compared to the semantic meaning conveyed by the ad. To conclude, despite expressing unpleasant emotions, commercials referring to the highly emotional pandemic experience may benefit the advertising efficacy, increasing the capability to reach customers.

## Introduction

Two years ago, the outbreak of COVID-19 pandemic entirely subverted the world we knew. Since then, many of our private, social, and cultural habits—and our believes—have radically changed, some of which will perhaps find a stable place in our new normality (He and Harris, 2020).

Among them, consumer behavior has been much affected by the pandemic situation, partly because of the world-wide supply-chain disruption, but also because of a range of psychological and emotional processes induced by the pandemic. COVID-19 has severely impacted on mental health around the world, increasing rates of depressive and anxiety symptoms, distress, and psychological diseases (Brooks et al., 2020; Casagrande et al., 2020; Huang and Zhao, 2020). The spreading of COVID-19 has disrupted in several ways the individuals’ perception of ontological security, jeopardizing the sense of order, and continuity about one’s own life and leading individuals to experience lack of control (Campbell et al., 2020).

Recent research has highlighted consumers may respond to the health and economic threats introduced by COVID-19 by strategically modifying their consumer behavior to reduce the perceived uncertainty. For instance, when exposed to contagion-related cues, people show a clear preference toward more authentic (i.e., perceived as traditional and genuine, such as a pizza based on the traditional Italian recipes passed on in a family restaurant compared to a foreign interpretation of the Italian pizza recipes; Park et al., 2021) or more familiar products (Galoni et al., 2020) because their outcomes are more predictable, which satisfies the consumers’ psychological need for security. Also, infection-related threats may lead consumers to variety-seeking behavior (Kim, 2020) and preference for atypical products (Huang and Sengupta, 2020) because of the motivation to restore control, freedom, and to reduce contagion probability. In addition, threat perception, uncertainty, and fear may also trigger panic buying behaviors (Campbell et al., 2020; Wang et al., 2020), such as hoarding and stockpiling of both sanitary (i.e., products that help facing the health threat, such as hand-sanitizers and masks) and comforting products (such as comfort food), which have been suggested to help consumers to recover the perception of control (Galoni et al., 2020).

Beyond modifications in consumers’ preferences, special attention should be paid to the way brands’ communications are perceived by consumers under the heavy emotional legacy of COVID-19 threat and to how consumers’ relationship with brands may be influenced by such uncertain circumstances. Indeed, a common practice among marketers is to adopt impactful emotional appeals in advertising campaigns, under the assumption that the higher the emotional impact, the more memorable and effective the appeal (Das et al., 2015; Akram et al., 2018; Wu and Wen, 2019; Yoon and Lee, 2019). The pandemic period was no exception, with many brands undertaking *expressive* and *poetic* types of campaigns, rather than adopting *informational* strategies, in the attempt to reach consumers more effectively (Jiménez-Sánchez et al., 2020). However, the profound modification of the shared emotional baggage and of psychological wellbeing brought by COVID-19 on a large part of the population should raise concerns about the use of extreme emotional appeals, especially when negative emotions are displayed. Under difficult circumstances, consumers were shown to be more responsive to affective inputs (Faraji-Rad and Pham, 2017). As a result, emotions could be perceived more intensely than usual, causing unnecessary distress to the consumers (Mensa and Vargas-Bianchi, 2020). Notably, some scholars have highlighted that emotions passively experienced though ads (i.e., *esthetic* emotions) are distinct from emotions evoked by real situations (i.e., *utilitarian* emotions) in that esthetic emotions are not strong enough to evoke the entire spectrum of emotions but rather can only elicit an appraisal in terms of hedonic pleasure or displeasure (Lajante et al., 2020). However, this distinction does not prevent appeals from eliciting significant subjective feelings that can nonetheless impact the consumer’s emotional sphere as long as the ads are vivid enough, according to the *law of apparent reality* (Frijda, 1986). For this reason, a careful evaluation of emotional tones adopted in ads should play an important role in these times, also considering that the emotional content can significantly affect the formation of attitudes toward the brand and purchase intention (Heath et al., 2006; Lin, 2011; Leanza and Balconi, 2017; Lajante et al., 2020). Thus, pitfalls should not be underestimated. An example of the risks associated with emotionally framed communications can be seen in coverage of COVID-19 news by media during the emergency phase of the pandemic, which resulted in accentuating fear about contagion, anxiety, and uncertainty, causing severe concrete repercussions also on public health (Garfin et al., 2020; Ogbodo et al., 2020).

A long-standing debate divides the scientific community about the effectiveness of emotional advertisement impact on the consumers’ attitudes and behaviors. While positive appeals have long been recognized as a safe strategy for gaining the consumers’ attention and shaping their attitudes (Aaker and Bruzzone, 1981; Poels and Dewitte, 2008; Berger and Milkman, 2012; Wu et al., 2018; Li, 2019; Coleman et al., 2020), the attempts of using negative emotions in ads did not always result in effective outcomes. Guilt and fear often revealed to induce actual behavioral changes (Giachino et al., 2017; Coleman et al., 2020; Zheng, 2020) but, when emotions are over emphasized, the manipulative intent of the ad may become too blatant, inducing offense, annoyance, and avoidance in the consumer (Wolburg, 2006; Brennan and Binney, 2010).

Considering the conflicting findings presented by the extant literature on the use of emotional appeals, the outbreak of COVID-19 pandemic represents a unique opportunity to study the impact of highly emotional contents on brand communication and, consequently, on the eventual modification of the attitude toward the brand (Taylor, 2020). Indeed, during the pandemic, numerous COVID-19-themed audiovisual advertisements have been produced, from both governmental sources (mainly with informative purposes) and business companies (which made an extensive use of emotional contents to support StayHome and gratitude campaigns, encouraging messages or just to endorse the brand image; Jiménez-Sánchez et al., 2020). Despite a few studies have investigated how emotions were employed in advertisings during the first months of the emergency (Jiménez-Sánchez et al., 2020; Mensa and Vargas-Bianchi, 2020), to the best of the authors’ knowledge, no studies have examined the actual impact of COVID-19-related brand communications on consumer perception so far.

For this reason, the present study aims at sorting out how the complex emotions elicited by the COVID-19 stimuli can modulate the attitude toward the brand. Since prior studies have demonstrated that the emotional and motivational responses toward an ad or a brand could be reflected by an asymmetrical pattern of activation within the prefrontal cortex (PFC; Ohme et al., 2010; Vecchiato et al., 2011; Maison and Oleksy, 2017; Garczarek-Bąk and Disterheft, 2018; Duan et al., 2021), in the present study, the functional Near-Infrared Spectroscopy (fNIRS) was used to assess the neurophysiological correlates of the emotional and motivational responses produced by the viewing of commercials that could display some COVID-19-themed content or not. fNIRS was chosen because it has proven to be a well-suited neuroscientific tool that allows to record with optimal spatial resolution the hemodynamic modulation within the PFC and to get an accurate estimate of the prefrontal asymmetry index (Balconi et al., 2015, 2017; Duan et al., 2021). Specifically, we expect that the COVID-19-related content may induce higher emotional responsivity compared to the content non-related to COVID-19, leading to a higher activation of the prefrontal cortex (Mondino et al., 2015). Moreover, we derived our second hypothesis from the approach-avoidance model (Davidson, 2004) and the abundant consistent evidence showing that left-lateralized prefrontal activity is a neurophysiological marker of the appetitive motivational system, whereas right-lateralized activity is a marker of the inhibition system. Such cerebral activity is thought to reflect action tendencies and to be independent from emotional valence processing (Sutton and Davidson, 1997; Vecchiato et al., 2014). Hence, we expect that commercials perceived as attractive (regardless of their emotional valence) would generate approach motivations toward the brand and that such process would result into a higher recruitment of the left hemisphere. Conversely, we expect that unappealing commercials would generate avoidance motivations toward the brand and that such process would result into a higher recruitment of the right hemisphere.

## Materials and Methods

### Sample

A sample of 20 Italian healthy participants [age range 20–30 years old; Mean (*M*) age = 25.47; Standard Deviation (*SD*) = 2.25; 5 males] were recruited for this study. The presence of a neurological, psychopathological disorder, the assumption of psychopharmacological therapy, or the presence of post-traumatic stress symptomatology linked to the COVID-19 experience (assessed through the COVID-19-PTSD questionnaire; Forte et al., 2020) constituted the exclusion criteria. Also, any severe medical or chronic condition, prior record of seizures, cranial injury, or chronic pain determined the exclusion of the participant from the experiment. All the included participants had a normal-to-corrected vision, were right-handed, and did not score significantly higher level of PTSD symptomatology at the COVID-19-PTSD. All the participants were college students that spent the COVID-19 emergency in northern Italy. They were recruited within the university campus of the Catholic University of the Sacred Heart of Milan, and they all signed a written informed consent from participants to take part in the experiment. They were informed no compensation was provided for their participation in the study. Participants were randomly assigned to two different groups that watched COVID-19-related or non-COVID-19-related commercials. The groups were balanced for age: COVID-19 group (*M* = 25.60, *SD* = 2.50) and non-COVID-19 group (*M* = 25.33, *SD* = 2.23). The research protocol has been implemented under the regulations of the Declaration of Helsinki (1964) and has been approved by the ethic committee of the Department of Psychology, Catholic University of the Sacred Heart of Milan, in Italy.

### Selected Advertising Stimuli

The set of stimuli consists of a total of six commercials belonging to the popular clothing brand of Nike with a strong mission toward Corporate Social Responsibility in terms of social promotion.

Three commercials were selected that refer to the COVID-19 theme: “Play for the World,” “You Cannot Stop LA,” and “You Cannot Stop Us.” The typical communication of this brand, full of emotional and motivational elements that aim at empowering and inspiring the audience, is intertwined with the narrative of today’s difficult historical period, characterized by the COVID-19 pandemic. These stimuli have been selected because they generally reflect the characteristics that have been adopted for the choice of stimuli in the work of Ohme et al. (2010); in fact, they can be distinguished in two frames: emotional frames, characterized by sequences of images with a high emotional impact; information frames, characterized by sequences of scenes in which there are mainly captions, emotional phrases, referring to the pandemic situation from COVID-19, superimposed images, and sequences where the brand logo is shown (Balconi and Lucchiari, 2005).

The storyline of the “Play for the World” commercial resumes the period of the first lockdown, in which people were forced to stay in home. Slow-motion images are taken of ordinary people and popular athletes who train inside the walls of the house, in the living room or in the corridor, and maintain social distancing. The commercial “You Cannot Stop LA” is dedicated to the Lakers basketball team, leader in the American NBA championship. The video sequences film the matches won and lost by the team which, symbolically, are accompanied by the most touching moments of the year, emphasizing the social victories and defeats associated with the pandemic. The plot of “You Cannot Stop Us” resumes the post-lockdown period from the COVID-19 pandemic. The peculiarity of the spot is the mix of images of sports team matches, followed by those of empty stadiums, canceled matches, and people training at home. The following scenes, symbolically, celebrate the end of this lockdown, emphasizing the return to sport and open-door sports competitions.

The following three non-COVID-19-related commercials, which do not make use of contents relating to the emergency healthcare from COVID-19 but are still characterized by emotional, motivational, and inspirational elements, were selected as: “What is your motivation?,” “You cannot be stopped,” and “Steps”. The plot of “What is your motivation?” describes the story of a young basketball player reminding its viewers that victory does not come from nowhere, but it requires lots of training. The commercial “You cannot be stopped” intends to help athletes unearth and tap into the true reasons that motivate us, our whys, for the year ahead. The “Steps” commercial is a motivational and emotional story of the journey of running while using Nike’s brand.

### Validation of the Stimuli

The stimuli were evaluated for the following perceptual characteristics: duration, fps, size, brightness, and content. The duration of the selected spots was 60 s, with 24 fps each, and they were validated for the content. A pool of independent judges controlled for gender and age, assessed the emotional valence and arousal of the stimuli using an adapted 5-point version of the Self-Assessment Manikin (SAM) scale and the 7-point semantic differential (Bradley and Lang, 1994). At the SAM, the selected COVID-19-related ads stimuli were rated higher for emotional valence (*M* = 4.17, *SD* = 0.89) and arousal (*M* = 3.83, *SD* = 1.07) such as non-COVID-19-related videos’ emotional valence (*M* = 4, *SD* = 0.95) and arousal (*M* = 3.33, *SD* = 1.10). While for the semantic differential scale, the average of the seven main characteristics are reported for the COVID-19-related videos (understandable: *M* = 6.00, *SD* = 1.15; familiar: *M* = 4.17, *SD* = 1.95; exciting: *M* = 6.17, *SD* = 1.21; engaging: *M* = 5.83, *SD* = 1.34; joyful: *M* = 5.50, S*D* = 1.12; motivating: *M* = 6.17, *SD* = 0.89; and pleasant: *M* = 0.65, *SD* = 0.76) and non-COVID-19-related videos (understandable: *M* = 5.83, *SD* = 1.34; familiar: *M* = 4, *SD* = 0.58; exciting: *M* = 5.00, *SD* = 1.53; engaging: *M* = 4.83, *SD* = 1.34; joyful: *M* = 4.67, *SD* = 1.37; motivating: *M* = 5.50, *SD* = 1.12; and pleasant: *M* = 5.33, *SD* = 1.24). No significant differences were found about these features.

### Procedure

For the experimental phase, the participants were sitting in a comfortable chair in a darkened room, with the monitor screen about 80 cm in front of their eyes. The hemodynamic response at rest was recorded for an open-eyes baseline lasting 120 s at the beginning of the experiment. After the baseline, the participants assigned to the COVID-19 group watched the three COVID-19-related videos, while to the subjects assigned to the non-COVID-19 group the non-COVID-19-related advertisement was proposed. All the videos were presented in randomized order (separated by a 5 s inter-stimulus interval during which a black screen was displayed) in the center of the computer monitor during fNIRS recording. The whole experimental phase lasted less than 20 min.

### fNIRS Configuration

The hemodynamic signal was recorded considering oxygenated hemoglobin (O_2_Hb) and deoxygenated hemoglobin (HHb) concentration fluctuations using a 6-channel optodes matrix from a NIRScout System (NIRx Medical Technologies, LLC, Los Angeles, California). A fNIRS Cap was used to position four light sources/emitters and four detectors over the head in accordance with the standard international 10/5 system (Oostenveld and Praamstra, 2001; Balconi and Molteni, 2015).

**Figure 1:**
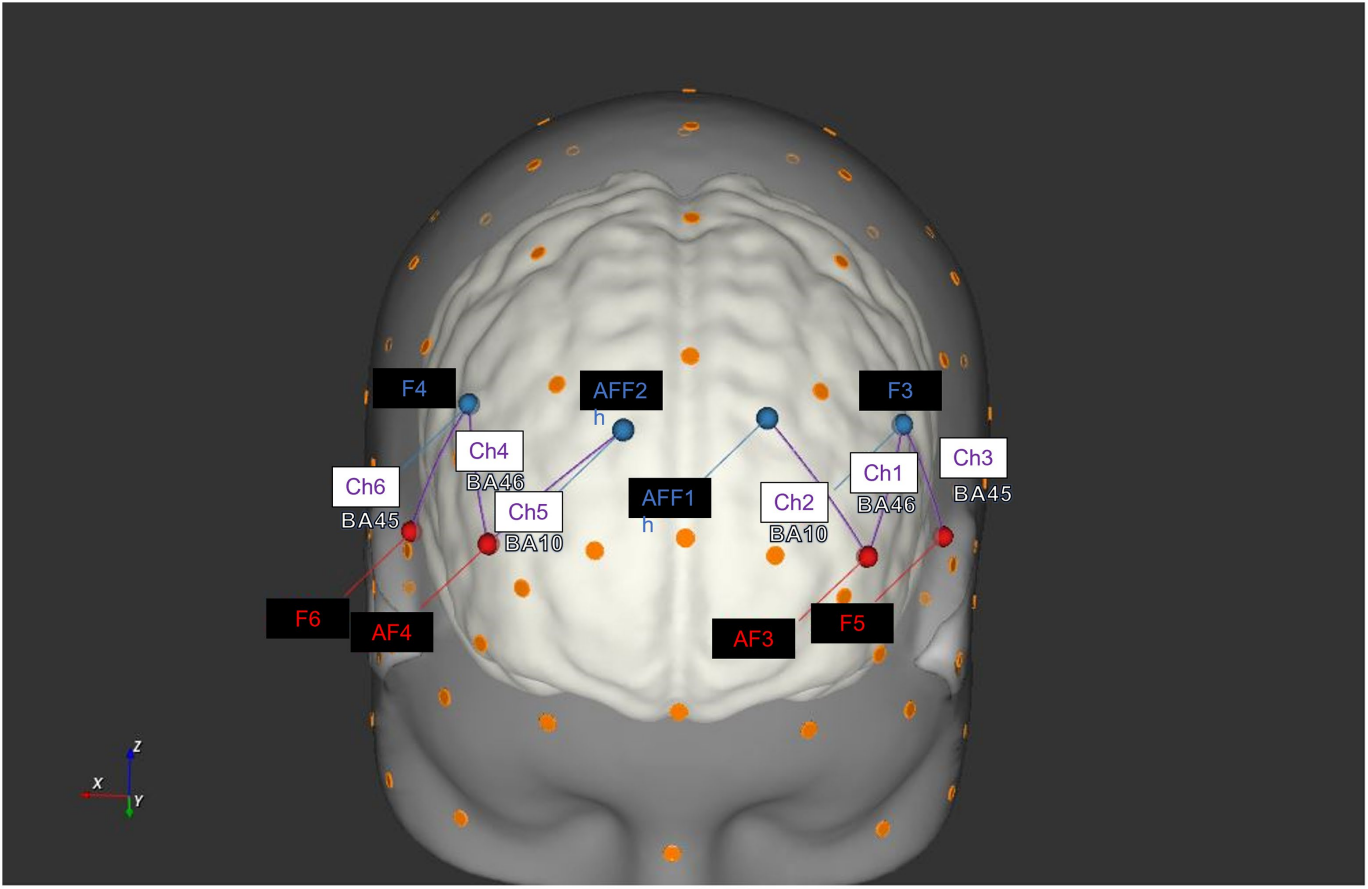
Configuration of fNIRS probes. The head rendering displays the position of the fNIRS sources (red) and detectors (blue). It has been generated with software NIRSite (NIRx Medical Technologies LLC) and then adapted with the correspondence between the channels (violet) and Brodmann areas is indicated in the figure: Ch1 and Ch4 correspond to left and right DLPFC (BA 46); Ch2 and Ch5 cover the left and right frontopolar area (BA10), and a portion of left and right DLPFC (BA 46); and Ch3 and Ch6 are consistent with the left and right pars triangularis Broca’s area (BA45).

Four emitters were placed in AF3-AF4, and F5-F6, and four detectors were installed in AFF1h-AFF2h, and F3-F4 in the device setup. The emitter-detector distance was kept constant at 30 mm for consecutive optodes, and near-infrared light with two wavelengths was used (760 and 850 nm). It was feasible to obtain a total of six channels with the following optode configuration: Ch1 (AF3-F3), Ch2 (AF3-AFF1h), Ch3 (F5-F3), Ch4 (AF4-F4), Ch5 (AF4-AFF2h), and Ch6 (F6-F4), consistent with previous studies (Balconi and Vanutelli, 2017; Balconi and Angioletti, 2021). To assign hemodynamic response alterations during the task to a specific brain region, it was adopted a probabilistic atlas implemented in the software fOLD [fNIRS Optodes’ Location Decider (Zimeo Morais et al., 2018)] using the automated anatomical labeling atlas Brodmann (Rorden and Brett, 2000). The correspondence between fNIRS channels and Brodmann areas is here reported: Ch1 and Ch4 correspond to left and right DLPFC (BA 46); Ch2 and Ch5 cover the left and right frontopolar area (BA10), and a portion of left and right DLPFC (BA 46); and Ch3 and Ch6 are consistent with the left and right pars triangularis Broca’s area (BA 45; Figure 1).

### fNIRS Biosignal Analysis

**Figure 2:**
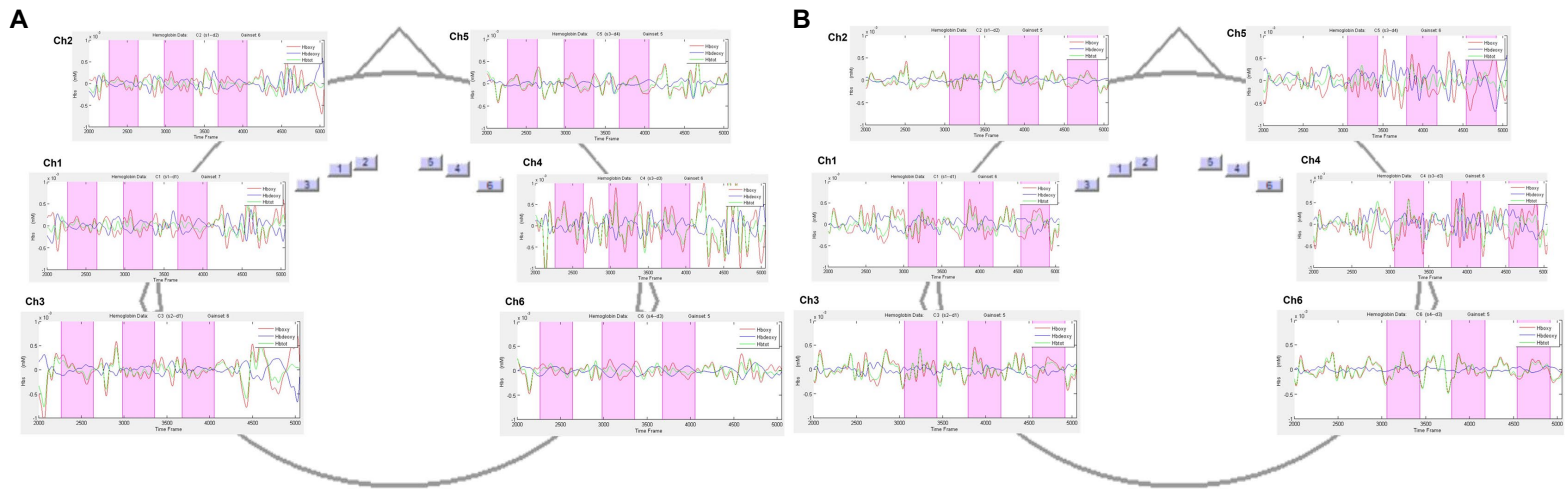
Hemodynamic signal time course for all channels under the two conditions. The figure shows the time course plots of O_2_Hb and HHb signal for each channel when watching the three commercials of the **(A)** COVID-19-related condition and **(B)** non-COVID-19-related condition.

By employing NIRStar Acquisition Software, the fluctuations in O_2_Hb and HHb were measured continuously through the task, starting with a 120 s resting baseline. A sample rate of 6.25 Hz was used to collect the signal deriving from the channels. The biosignal was analyzed employing nirsLAB software (v2014.05; NIRx Medical Technologies LLC, 15Cherry Lane, Glen Head, NY, United States), based on their wavelength and position, yielding mmol^*^mm values corresponding to changes in O_2_Hb and HHb concentrations per channel. A digital band-pass filter at 0.01–0.3 Hz was adopted to filter the biosignals obtained from each channel. Figure 2 shows the plots of the time course for all channels under the two conditions: COVID-19-related (Figure 2A) and non-COVID-19 (Figure 2B). For each channel, the average O_2_Hb and HHb were calculated for the task. The average concentrations in the time series for each channel and individual were used to calculate the effect size in each condition. The following formula was adopted to calculate the effect sizes (Cohen’s d). They were calculated by dividing the difference between the baseline and trial means by the baseline standard deviation (SD): D = (m1–m2)/s, where m1 and m2 represent the baseline and trial mean concentration levels, respectively, and s represents the baseline SD. The effect sizes from the six channels were averaged to improve the signal-to-noise ratio. Unlike raw fNIRS data, which were originally relative values that could not be directly averaged across subjects or channels, normalized effect size data were averaged regardless of the unit because effect size is unaffected by differential pathlength factor (DPF).

### Statistical Data Analysis

Two repeated-measures ANOVAs with independent between factor *Group* (2: COVID-19, non-COVID-19) and within factor *Channel* (6: Ch1, Ch2, Ch3, Ch4, Ch5, and Ch6) were performed on D dependent fNIRS data (O_2_Hb and HHb mean values). Pairwise comparisons were applied to check simple effects for significant interactions, and Bonferroni correction was used to eliminate potential biases in repeated comparisons. Greenhouse–Geisser epsilon was used to adjust the degrees of freedom for all ANOVA tests. In addition, the kurtosis and asymmetry indices were adopted to determine the normality of the data distribution. By estimating partial eta squared (*η*^2^) indices, the size of statistically significant effects has been assessed.

## Results

The statistical analyses performed on D dependent measures for O_2_Hb and HHb concentration means yielded the following evidence.

**Figure 3:**
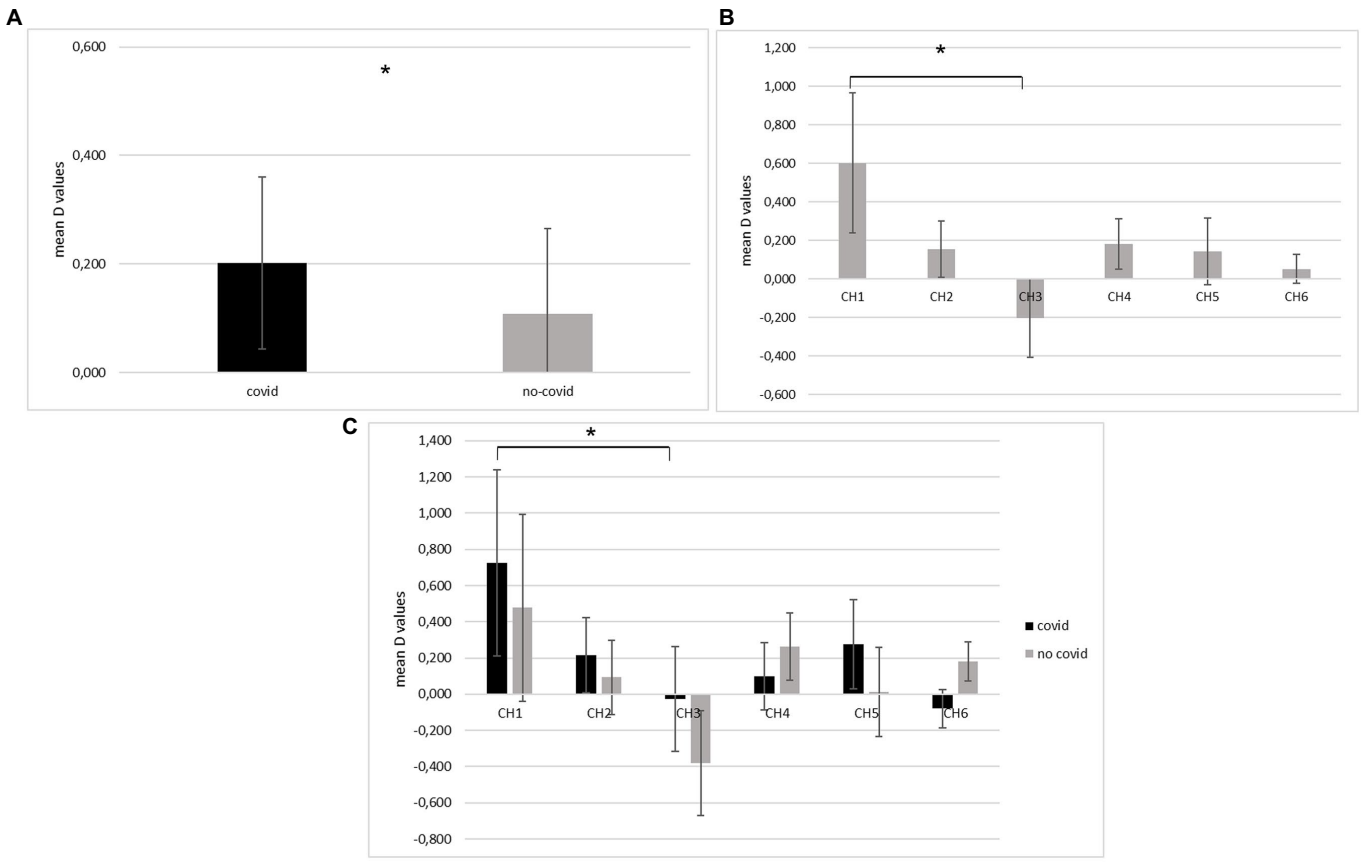
Hemodynamic O_2_Hb results. **(A)** The graph displays O2Hb modulation (D values) as a function of Group, which is significantly increased for the COVID-19 group compared to the non-COVID-19 group. **(B)** The bar chart shows significantly higher O_2_Hb values in the first channel (Ch1), corresponding to left DLPFC (BA46), compared to the third channel (Ch3) consistent with the left pars triangularis Broca’s area (BA45) for all participants. **(C)** As the bar graph shows, significantly greater mean O_2_Hb values were found in the Ch1 (left DLPFC) than Ch3 (left pars triangularis Broca’s area) in the COVID-19 group. All data are represented as mean ± SE; all ^*^statistically significant differences, with *p* ≤ 0.05.

About O_2_Hb, a first significant main effect for the *Group* was observed [*F*(2, 18) = 6.75, *p* = −01, *η*^2^ = 0.387] (Figure 3A).

Secondly, a significant main effect was found for the *Channel* for the O_2_Hb [*F*(5,18) = 6.04, *p* = 0.01, *η*^2^ = 0.311]. In particular, as revealed by pairwise comparisons, greater neural responsiveness (increase of O_2_Hb) was found in Channel 1 compared to Channel 3 [*F*(1,18) = 7.98, *p* = 0.01, *η*^2^ = 0.398] (Figure 3B).

Thirdly, a significant interaction effect *Group* × *Channel* was detected for O_2_Hb values [*F*(5,18) = 6.09, *p* = 0.01, *η*^2^ = 0.365]. Specifically, pairwise comparisons showed significant higher mean values in the Channel 1 compared to Channel 3 for the COVID-19 group [*F*(1,18) = 8.09, *p* = 0.01, *η*^2^ = 0.412] (Figure 3C).

For the HHb mean values, no significant effects were observed.

## Discussion

The present study aimed at investigating the impact of highly emotional, COVID-19-related, and brand communications on consumer attitudes toward the brand. First, the findings suggest that the COVID-19-related contents were able to prompt emotional processing within the PFC to a higher extent with respect to contents non-related to COVID-19. Indeed, overall, an increase in the levels of O_2_Hb was found within the PFC for individuals who were exposed to COVID-19-related advertisings compared to advertisings unrelated to COVID-19 pandemic. This result is consistent with prior studies showing that PFC—specifically, the dorsolateral portion (DLPFC)—plays a relevant role in processing emotion information (Balconi et al., 2011; Balconi and Bortolotti, 2012; Mondino et al., 2015), orienting attention toward emotional stimuli (Steele and Lawrie, 2004; Jacob et al., 2014), and regulating the emotional valence, while ventromedial PFC is involved in emotion regulation and control of emotional arousal (Etkin et al., 2011; Nejati et al., 2021). Previous studies have also highlighted that the dorsomedial PFC is specifically engaged by the appraisal and the expression of emotions, with an abundance of evidence for negative emotions processing (Mechias et al., 2010; Etkin et al., 2011). Thus, our results suggest that a more intensive emotional processing may be triggered by the social communication conveyed by COVID-19-related advertisings and that the experiential recall of a shared emotional experience may impact on the effectiveness of the content and be successful in reaching the consumers, despite also conveying some negative emotions together with positive motivational contents.

Secondly, our results reveal that, overall, the left DLPFC was more active than the left Broca pars triangularis (BA 45) across the whole range of advertisings. However, this effect was particularly evident when participants saw the COVID-19-themed contents. The literature has provided generous evidence of the specialization of left DLPFC in the processing of pleasant emotional stimuli eliciting approach motivation, as opposed to the role of right DLPFC in the responsivity to negative emotional stimuli, eliciting avoidance motivation (Davidson, 2004; Herrington et al., 2005; Balconi and Ferrari, 2012; Mondino et al., 2015; Roesmann et al., 2019). Notably, it is possible that such imbalance between the left and right frontal activity does not reflect the valence *per se*, but rather it could reflect the approach-avoidance motivation triggered by a stimulus, irrespective to its valence (Sutton and Davidson, 1997). On the other hand, the left inferior frontal gyrus (IFG, BA 45) has been consistently associated to the processing and the selection of semantic information (Grindrod et al., 2008; Guenther et al., 2015). Taken together, the evidence from the extant literature may lead to hypothesize that the commercials used in the present study may have stressed out the expressive and emotional aspect more than the verbal content, driving our participants to dedicate more attention toward the processing of the emotional components compared to the semantic meaning conveyed by the ad, and eliciting an approach motivation. Furthermore, it is possible that COVID-19-related contents were more effective compared to COVID-19-non-related contents in orienting the consumers’ attention toward emotional information rather than toward the elaboration of the semantic meaning of the claim, resulting in a deeper engagement on the emotional processing and, consequently, in a stronger recruitment of the left DLPFC. However, because the IFG was also shown to be implicated in emotional empathy and—in case of injury—alexithymia (Shamay-Tsoory et al., 2009; Hobson et al., 2018), an alternative hypothesis is that the contrasting emotional content was so much intense to make our participants struggle to recruit the emotional contagion system served by the IFG, which resulted being engaged to a lesser extent. Future studies may further explore the reason for such effect, perhaps delving more specifically into the possibility that an excessive use of emotional content may backfire, inducing undesirable outcomes on consumer response (Wolburg, 2006). Relatedly, indeed, sustained mental stress was shown to decrease oxygenated hemoglobin concentrations (i.e., to decrease activity) within the right PFC, which is considered to be consistent with an increased left-lateralized pattern of activity and related to the frontal asymmetry (Al-Shargie et al., 2016, 2017).

In addition, future research could take into consideration the role of interindividual differences in sensitivity to cognitive vs. emotional aspects in the fruition of COVID-19-related appeals. Indeed, Aquino et al. (2020) showed that commercials that match the participants’ personal orientation in terms of their Need for Affects or Need for Cognition are perceived as more relevant, specifically recruiting to a higher extent the ventromedial prefrontal cortex (vmPFC). Future studies then could investigate whether people with a stronger orientation toward affect could be more persuaded by strong emotional contents referred to the pandemic scenario.

In conclusion, the present study has shed some light on the use of highly emotional contents containing references to a shared impactful negative experience (i.e., COVID-19 pandemic) presented with a positively framed, expressive, and motivational tone. The present findings suggest that, if cleverly designed, COVID-19-related commercials could elicit more intense emotional responses and modulate the perception of the ad, potentially acting as a lever to increase consumer appeal. The pandemic content, indeed, could serve as a natural source of engagement for potential customers. Moreover, these results could be generalized to other high-impact stimuli of a different nature than the pandemic scenario. However, some limitations should be considered in the present study. The study did not collect any behavioral measure to assess behavioral attitude changes nor explicit judgments following the experimental manipulation. Future research will need to further investigate if such commercial strategy is also capable of effectively changing the perception of the brand, enhancing the consumer attitude toward the brand and, eventually, increasing their willingness to buy products from the advertised brand. Secondly, emotional responses were only evaluated according to neurophysiological markers, whereas explicit emotional evaluations were not taken into account. Third, future research may benefit from a within-participants design so that, by exposing each participant to both COVID-19-related and non-related contents in a counterbalanced order, it would be possible to control for the effect of interindividual differences related to the pre-existing preference for the brand or the degree of previous exposure to the COVID-19 threat. Also, as emotional processing is not only subserved by the PFC, assessing the contribution of other cortical sites may prove constructive to achieve a deeper understanding. Finally, the results presented in this study should not be interpreted as conclusive, since the limited sample size may have lowered the power of the statistical tests, offering a partial appreciation of the COVID-19 communication effects. Future studies should consider recruiting a larger sample size, following the requirements suggested by a power analysis, to produce statistically stronger results.

## Data Availability Statement

The raw data supporting the conclusions of this article will be made available by the authors, without undue reservation.

## Ethics Statement

The studies involving human participants were reviewed and approved by the Ethic Committee of the Department of Psychology, Catholic University of the Sacred Heart of Milan, Italy. The patients/participants provided their written informed consent to participate in this study.

## Author Contributions

MB and MS contributed to the conception and design of the study. MS and LA wrote the first draft of the manuscript. MB, MS, and LA contributed to the manuscript final writing and revision, read, and approved the submitted version. All authors contributed to the article and approved the submitted version.

## Conflict of Interest

The authors declare that the research was conducted in the absence of any commercial or financial relationships that could be construed as a potential conflict of interest.

## Publisher’s Note

All claims expressed in this article are solely those of the authors and do not necessarily represent those of their affiliated organizations, or those of the publisher, the editors and the reviewers. Any product that may be evaluated in this article, or claim that may be made by its manufacturer, is not guaranteed or endorsed by the publisher.
